# Clinical and treatment profile with five-year survival analysis of colorectal cancer from Himalayan state of India

**DOI:** 10.3332/ecancer.2025.1979

**Published:** 2025-08-29

**Authors:** Kaushal Singh Rathore, U K Chandel, Niharika Singh

**Affiliations:** 1 Department of Surgical Gastroenterology, All India Institute of Medical Sciences, Jodhpur 342001, India; 2 Department of General Surgery, Indira Gandhi Medical College, Shimla 171001, India; 3 Department of General Medicine, All India Institute of Medical Sciences, Jodhpur 342001, India

**Keywords:** colorectal cancer, clinicopathological and treatment profile, follow-up, multimodality treatment

## Abstract

**Background:**

The incidence of colorectal cancer has been increasing worldwide. Middle- and low-income countries are also experiencing surge. This is due to a rapidly growing population.

**Methods:**

All patients diagnosed and presenting to Indira Gandhi Medical College, Shimla, the sole tertiary cancer care centre in Himachal Pradesh, India, during the study period from January 2017 to December 2018, were included in the study and were followed till 60 months. Data were collected on clinical characteristics, pathology, treatment received and survival outcomes, specifically overall survival (OS) and disease-free survival (DFS). A Kaplan–Meier survival curve was constructed and multiple regression analysis was conducted to identify factors influencing survival, with a significance level set at *p* < 0.05.

**Results:**

A total of 165 patients participated in the study, with a predominance of male subjects, a median age of 60 years and 34% of participants being under 50 years of age. The most prevalent symptoms were anorexia and weight loss, affecting 97% of the cohort, with the rectum identified as the most common site of involvement in 42% of cases. Histopathological analysis revealed signet ring-type morphology in 15% of the cases. The majority of patients presented with advanced stages, specifically stage 3 (36%) and stage 4 (32%). A multimodal treatment strategy was employed, involving collaboration among radiation oncologists, surgeons and medical oncologists. After a follow-up period of 60 months, 22 patients were lost to follow-up and only 23 patients remained alive. Multiple regression analysis indicated that only the stage of the disease significantly influenced overall survival (OS) and DFS.

**Conclusion:**

Limited health awareness, coupled with the challenging topography of the region, has led to a significant number of defaulters. This, in conjunction with the advanced stage of the disease and inadequate access to healthcare, has contributed to suboptimal OS and DFS rates. Further epidemiological investigations, including genetic analyses, are necessary to better characterise the presentation of this disease.

## Introduction

Colorectal cancer (CRC) ranks as the second leading cause of cancer-related mortality globally and is the third most prevalent cancer among both sexes [1, 2]. There is a notable global increase in both the incidence and prevalence of CRC, particularly in low- and middle-income countries (LMICs). However, data from high-income countries, especially those with established screening programs, indicate a stabilisation in CRC rates [3]. In India, CRC is on an upward trajectory, currently representing the seventh most common cancer among both sexes, with approximately 80%–90% of cases diagnosed in individuals over 50 years of age [4]. A recent update from the United States Multi-Society Task Force has lowered the screening age for average-risk individuals to 45 years, in response to the increased disease burden in those under 50 years [5]. The rising incidence in LMICs is attributed to factors such as increased obesity rates and dietary changes, including higher consumption of fast food and red meat, coupled with reduced dairy intake. Over the past two decades, many Asian countries have experienced a 2–4-fold increase in CRC incidence [6]. Globally, the prevalence of CRC in individuals under 40 years is approximately 2%–3%, yet reports from India suggest a significantly higher prevalence.

According to the Global Burden of Disease India study report (1990–2016), stomach cancer accounted for 9%, colon and rectum cancer for 5.8%, esophageal cancer for 4.3% and liver cancer for 3.5% of the cancer burden [7]. In India, the National Cancer Registry Programme was initiated in 1981 with the establishment of three population-based cancer registries, which have since expanded to 48, covering 16.5% of the population. The highest cumulative lifetime risk of gastrointestinal cancer was observed in India’s northeast region, ranging from 1 in 7 in the Aizawl and Papumpare districts to 1 in 49 in Manipur. In contrast, the western registries reported a range from 1 in 30 in Mumbai to 1 in 97 in Osmanabad and Beed. Aizawl also recorded the highest incidence of colon cancer (Males: AAR 7.2, Females: AAR 5.7) and rectal cancer (Males: AAR 8.6, Females: AAR 5.7) [8]. The clinical presentation of CRC is varied, including symptoms such as altered bowel habits, bleeding per rectum, anaemia and generalised weakness, as well as surgical emergencies due to perforation, obstruction and bleeding. Although hospital-based cancer registries have inherent selection biases, in the absence of population-based cancer registries in the Himalayan region of India, this epidemiological data serves as a crucial source of information for understanding the disease pattern in the region. This study aimed to investigate the clinicopathological and treatment profiles of CRC patients presenting at this tertiary care centre over a 2-year period, with a secondary endpoint of 5-year survival and factors affecting survival.

## Methods

This prospective observational study was conducted within the Department of General Surgery, in collaboration with the Departments of Pathology and Radiation Oncology, at Indira Gandhi Medical College (IGMC), Shimla, Himachal Pradesh. This institution serves as the sole regional cancer centre for the entire state of Himachal Pradesh. The study included all newly diagnosed CRC patients presenting from January 2017 to December 2018. Exclusion criteria encompassed patients who declined to provide consent and those with recurrent cases diagnosed or treated prior to January 2017. A prospective database was established to systematically record data related to demographics, clinical profiles, investigations, histopathology, staging, treatment details and survival outcomes. Each patient underwent a comprehensive history and physical examination, followed by investigations to confirm the diagnosis and stage of the disease. The study utilised a questionnaire, routine blood tests, CEA levels, colonoscopy, ultrasonography, contrast-enhanced computed tomography, magnetic resonance imaging and histopathological reports. The parameters examined included age, sex, lesion site, clinical presentation, histopathology of the lesion, disease stage, treatment received, factors influencing survival and 5-year survival rates.

### Treatment

All patients received treatment in accordance with the clinical stage of their disease. Resection was deemed curative if there was no preoperative evidence of metastasis and an R0 resection was confirmed by the histopathological report. All other resections were classified as palliative. Standard long-course neoadjuvant chemoradiotherapy for locally advanced rectal carcinoma and 5-Fluorouracil-based adjuvant chemotherapy were recommended in accordance with institutional guidelines.

### Follow up

All patients were monitored following their hospital visit, with annual telephonic follow-ups conducted to comprehensively document their health status. In the event of a patient’s death, the date of death was recorded by their relatives. The final follow-up occurred on December 31, 2023.

### Statistical analysis

All statistical analyses were conducted utilising SPSS software (version 21.0, SPSS Inc., Chicago, IL, USA). Numerical data are presented as mean, median, range, frequency and percentage, while categorical variables are expressed as frequencies and percentages. Overall survival (OS) was defined as the duration from the month of diagnosis to the month of death, irrespective of cause. Disease-free survival (DFS) was defined as the interval from the date of surgery to tumour recurrence or death. The final follow-up occurred on December 31, 2023. Patients who were lost to follow-up were excluded from the analysis and Kaplan–Meier survival curves were constructed. Statistical differences in the survival curves were assessed using the log-rank test. Multiple regression analysis was employed to identify factors associated with poor OS and DFS. A *p*-value of < 0.05 was considered indicative of statistical significance for all analyses.

### Definition of variables

The age at diagnosis refers to the age at which a patient presents at IGMC, Shimla. Patients diagnosed with CRC before the age of 40 are classified as having young-onset CRC, while those diagnosed after the age of 40 are classified as having late-onset CRC [9]. The Asian-Pacific Body Mass Index (BMI) classification was employed to assess nutritional status, with a BMI of less than 18.5 indicating malnourishment and a BMI greater than 23 indicating obesity. A positive family history of CRC is defined by the presence of the disease in family members, including spouses, first-degree relatives (siblings, children and parents) and second-degree relatives (half-siblings, grandchildren and grandparents). The AJCC 8th edition was utilised to determine clinicopathological variables [10]. Histological grades were determined according to the World Health Organisation 2019 guidelines [11]. Anatomically, the region from the cecum to the transverse colon is considered the right side, while the region from the splenic flexure to the rectosigmoid is considered the left side, extending beyond rectosigmoid into the rectum. Patients who did not attend follow-up appointments or could not be contacted were classified as lost to follow-up, and those who did not adhere to the treatment plan were classified as defaulters.

## Results

During the study period, a total of 267 patients presented with CRC. Of these, 96 cases were recurrences of previously diagnosed and treated patients and 6 patients did not provide consent, resulting in the inclusion of 165 patients in the study. CRC patients accounted for approximately 3.5% of the total cancer patients and represented the fifth and sixth most common cancers among males and females, respectively, at our centre. CRC occurrence in younger individuals was 16%, with the majority of cases observed in individuals over 50 years of age and the median age being 60 years. The most prevalent behavioural attribute associated with CRC was a non-vegetarian diet (consumption of non-vegetarian meals more than three times a week) in 75% of patients, followed by smoking in 54% of patients. Constitutional symptoms such as anorexia and significant weight loss (greater than 10% of body weight in the last 3 months) were observed in 96% of patients. The rectum was the most common site of CRC, present in 42% of cases, with well-differentiated adenocarcinoma being the most common histological type (Table 1).

**Figure 1.**
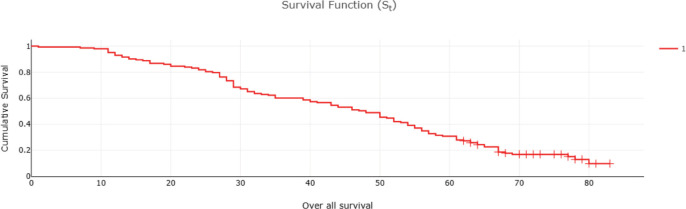
OS of CRC patients (months).

**Figure 2.**
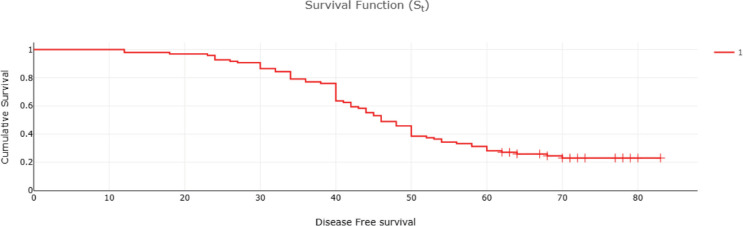
DFS of CRC patients (months).

**Figure 3.**
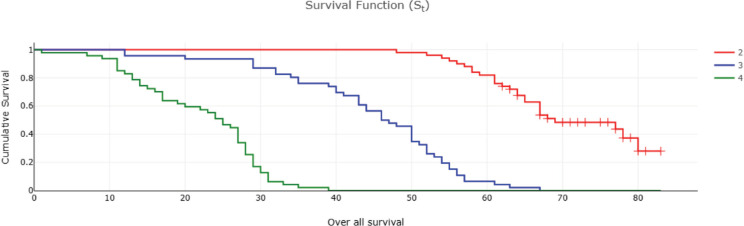
OS of CRC patients according to stage (months) 2- stage 1+2 , 3- stage 3, 4- stage 4.

**Figure 4.**
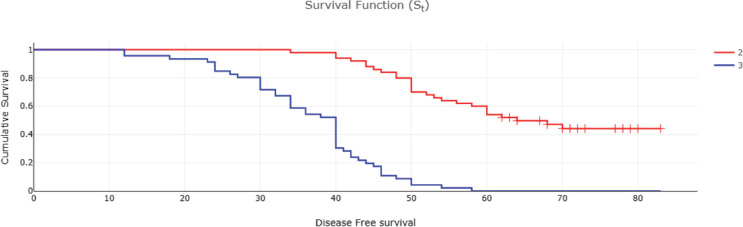
DFS of CRC patients according to stage 2-stage 1+2 , 3-stage 3.

**Figure 5.**
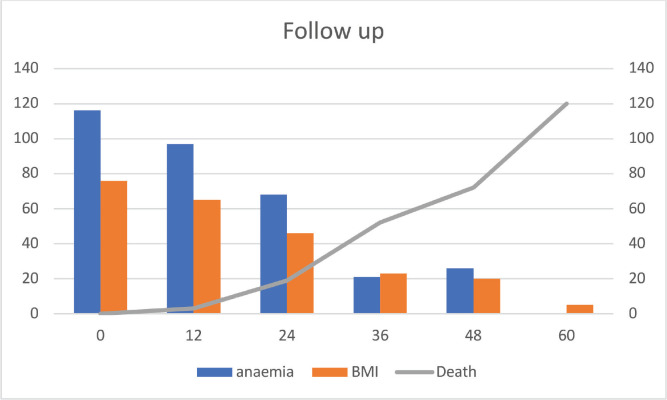
Follow up data on anaemia, BMI, death (N-143).

Fifty-two (32%) of CRC patients presented with stage 4 disease, with the liver being the most common site of metastasis. Isolated liver metastasis was observed in 15 (28.8%) patients. A multimodal treatment approach was employed, including both open (86%) and laparoscopic (14%) surgeries, emergency curative resection (10%) and palliative surgery (8.48%). Long-course neoadjuvant chemoradiotherapy was administered to 22% of patients. Eighty-two (50%) patients defaulted on treatment. At the conclusion of the follow-up period, approximately 13% were lost to follow-up and 14% were alive (Table 2). Among patients with stage I disease, 87% were alive, while none survived in stage IV (Table 2). The 5-year OS rate was 31% and the DFS rate was 28.7% (Tables 2 and 3). The median OS across all stages was calculated to be 48 months, with survival for stages 1–3 being 57 months and for stage 4 being 25 months (Table 4 and Figures 1 and 2). Kaplan–Meier curves indicated significantly better OS and DFS in patients with lower-stage disease (*p* <0.001) (Figures 3 and 4). Multiple regression analysis revealed that, apart from clinical stage, no other factor was significantly associated with OS and DFS (Tables 5 and 6). Follow-up data indicated improvements in BMI and anaemia status among CRC patients (Table 7 and Figure 5).

## Discussion

According to GLOBOCAN 2022, lung cancer is the most commonly newly diagnosed cancer, with CRC ranked third at 9%. The incidence of CRC at our centre is similar to the national average, at 3.5% [1]. Our hospital is the largest and only tertiary care cancer centre in the state, receiving referrals from distant regions. The median age of patients was 60 years, with a range of 21–91 years, which is higher than the findings of Patil *et al* [12] and similar to studies from Saudi Arabia and Singapore [13, 14]. One of the most significant factors is advanced age, as 66% of patients were over 50 years old. Patients under 50 years constituted approximately 35%, which is a matter of concern as it is considerably higher than reports from Western countries, which indicate 7% [15]. However, this is consistent with a report from India stating that CRC in individuals under 40 years is 35%–40% [16]. In addition to genetic and lifestyle factors, such as the westernisation of diet, another reason in India could be the growing population with a broad age pyramid; according to the 2011 census, 62.5% of the population falls within the age group of 15–59 years, unlike the West, which has a larger elderly population. Currently, there is no recommendation for CRC screening in India. However, in the near future, India will require screening guidelines to match the pace at which CRC incidence is rising, with special emphasis on issues such as psychosexuality, fertility and quality of life.

Consistent with several prior studies, a predominance of male participants was observed [17, 18]. This may be attributed to the prevalence of high-risk behaviours among males, such as smoking, consumption of processed and red meat and alcohol intake. Upon assessing behavioural attributes, it was found that 75% of participants consumed a non-vegetarian diet, 54% were smokers or tobacco chewers and 49% consumed alcohol. These findings align with a study from Indonesia, which demonstrated a strong association between smoking, a non-vegetarian diet and CRC [19]. Although obesity is commonly associated with the development and progression of CRC, our study revealed that the majority of patients were malnourished (54.5%), as most presented at an advanced stage. This contrasts with findings from Western countries, where obesity is more prevalent among CRC patients [20].

The clinical presentation of CRC patients is diverse, ranging from asymptomatic cases identified through screening colonoscopy to instances requiring emergency hospitalisation due to perforation and obstruction, with gastrointestinal haemorrhage occurring infrequently. A retrospective cohort study involving 29,000 patients identified the most prevalent presentation as a change in bowel habits and rectal bleeding [21]. In our study, the predominant presenting symptoms were anorexia and significant weight loss (exceeding 10% of body weight within 3 months), as well as anaemia, observed in 97% and 82% of patients, respectively. Retrospective inquiries revealed that a substantial proportion of patients (85%) experienced altered bowel habits; however, many disregarded these symptoms, opting for traditional medications and local treatments and only sought medical attention when the disease had progressed significantly. This pattern clearly indicates a low level of health literacy and awareness regarding CRC within the community.

The rectum was identified as the most prevalent site, accounting for 42% of cases, with left-sided tumours comprising 60%. Synchronous malignancy was observed in 5.5% of cases [17]. A significant proportion of patients exhibited signet ring cell morphology (15%), in contrast to Western reports, where this morphology is observed in approximately 1%–2% of cases [22, 23]. Of the 24 patients with signet ring morphology, five presented with stage IV disease, and all five (100%) exhibited diffuse peritoneal metastasis, indicating the aggressive nature of the disease. The majority of patients were diagnosed with Stage III (36%) and Stage IV (32%) disease, complicating treatment, increasing morbidity, prolonging treatment duration and consequently imposing a greater financial burden on the healthcare system [24, 25]. The liver was the most common site of metastasis (19%), followed by the lung (13%), consistent with the findings of Patil *et al* [12]. Liver-limited metastasis was observed in 15 (28.8%) patients, and no liver-directed therapies were administered to these patients.

Multimodal treatment was administered to patients based on their disease stage. Of these, 69% underwent curative surgery, which included laparoscopic surgery (10%) and emergency surgery (6%). Among the 24 patients who presented in an emergency setting, 14 underwent palliative surgery. This included the formation of a diversion stoma in 10 patients and palliative resection in 4 patients, due to tumour perforation and faecal peritonitis. Additionally, 22% of patients with rectal cancer received neoadjuvant chemoradiotherapy, while palliative chemotherapy was provided to 10.3% of patients [26].

SurvCan-3 reports the 5-year net survival rate (2008–2012) for CRC in India as 34.2% for colon cancer and 37.9% for rectal cancer [27]. In contrast, the 5-year net survival rate was 65% (2014–2020) in the United States and 58.4% (2016–2020) in England [28, 29]. Our 5-year OS is comparable to a recent study from Rwanda [30] and is lower than the 40% reported from a high-volume centre in Mumbai [12] and the 60% reported from a study in China [23]. When comparing the 5-year OS from a study in Delhi [31], the survival rates for stages I (88% versus 82%), II (83% versus 80%), III (7% versus 55%) and IV (0% versus 32%) indicate poor survival in stages III and IV. This disparity can be attributed to patient factors leading to high defaulter rates like financial constraints, illiteracy, lack of awareness, challenging terrain and harsh climatic conditions. Additionally, surgeon-related factors, such as the poor quality of surgical specimens with inadequate lymph node clearance and general factors, including poor nutritional status, lack of targeted therapy based on genomic analysis and unknown inherent genetic factors that result in poorly differentiated/signet-ring cell morphology cancers, particularly in the young population.

The 5-year DFS rate was 28%, and the Kaplan–Meier curve also demonstrated significantly poorer DFS with advancing stage. In a multiple regression analysis to identify factors influencing OS and DFS, only clinical stage was found to be associated with poorer OS and DFS. This finding contrasts with previous studies that have identified age, female sex and poor histology as predictors of poor survival [32]. In our region, there is a general consensus that surgery is the primary means to extend life in cancer patients. Consequently, when we recommended neoadjuvant therapy, all patients adhered to our advice. However, in the adjuvant or palliative settings, there were numerous instances of non-compliance. The median OS was 48 months, with a significant difference observed between the median survival of patients receiving treatment compared to those receiving palliative care (57 versus 25 months, *p* < 0.001). Additionally, stage emerged as a significant predictor of overall survival (*p* < 0.001) [26, 33].

The limitations of this study include its small sample size, descriptive methodology and the lack of evaluation of genetic factors influencing CRC in young individuals and poor survival rates. As this represents the first dataset from our Himalayan state in India, characterised by its hilly terrain, harsh climatic conditions and limited healthcare access, we anticipate that this study will contribute to program and policy development in India. This effort aims to reduce both the number of patients and the mortality rate, similar to the outcomes observed in Taiwan, where the implementation of a nationwide screening program resulted in a 15% reduction in mortality between 2014 and 2017 [34].

## Conclusion

The clinical trends of CRC in our region differ from those observed in Western countries, as a significant proportion of cases occur in individuals under the age of 40 and present at advanced stages. In terms of management, we lack expertise and do not provide liver-directed therapies, which are currently considered the standard of care. Our poor survival rates can be attributed to socioeconomic factors, the absence of comprehensive health policies, inadequate access to healthcare, challenging topography (hilly terrain) and limited expertise.

## List of abbrevations

CRC, Colorectal cancer; Well diff adenoca, Well differentiated adenocarcinoma; Mod diff adenoca, Moderately differentiated adenocarcinoma; Poor diff adenoca, Poorly differentiated adenocarcinoma; OS, Overall survival; DFS, Disease free survival.

## Conflicts of interest

Nothing to disclose.

## Funding

No financial disclosure.

## Author contributions

- Kaushal Singh Rathore: Substantial contributions to the conception, design of the work, analysis, interpretation of data, drafting the work and reviewing it critically for important intellectual content, final approval of the version to be published and agreement to be accountable for all aspects of the work in ensuring that questions related to the accuracy or integrity of any part of the work are appropriately investigated and resolved.
- UK Chandel: Substantial contributions to the conception, drafting the work and reviewing it critically for important intellectual content, final approval of the version to be published and agreement to be accountable for all aspects of the work in ensuring that questions related to the accuracy or integrity of any part of the work are appropriately investigated and resolved.
- Niharika Singh: Design of the work, analysis, interpretation of data, drafting the work and reviewing it critically for important intellectual content, final approval of the version to be published and agreement to be accountable for all aspects of the work in ensuring that questions related to the accuracy or integrity of any part of the work are appropriately investigated and resolved.Sudharshan K Sharma: Design of the work, analysis, interpretation of data, drafting the work and reviewing it critically for important intellectual content, final approval of the version to be published and agreement to be accountable for all aspects of the work in ensuring that questions related to the accuracy or integrity of any part of the work are appropriately investigated and resolved.

## Figures and Tables

**Table 1.** Demographic and treatment profile of presenting colorectal cancer patients.

Parameter	Number	Percentage
Age group(years) 21–30 31–40 41–50 51–60 61–70 >70	10 16 29 35 43 32	6.06 9.69 17.57 21.21 26.06 19.39
Median age (range) years	60 (21–91)	------
Gender Male Female	92 73	55.75 44.25
Co morbidity Hypertension Diabetes mellitus	13 12	7.87 7.27
Risk factors Smoker Alcohol Non vegetarian Family history	89 80 124 1	53.93 48.48 75.15 0.60
BMI <18.5(Malnourished) 18.5–23.5(Normal) >23(Obese)	90 41 34	54.5 24.84 20.60
Clinical presentation Pain abdomen Lump abdomen Haematochezia Melena Anaemia^*^ Anorexia and weight loss^$^ Altered bowel habits Emergency^#^	89 41 91 78 135 160 140 24	53.93 24.89 55.15 47.27 81.81 96.96 84.84 14.5
Tumour location Right Left Rectum Synchronous	58 29 69 9	35.15 17.57 41.81 5.45
Histopathology WDA MDA PDA Signet ring cell type Others	65 55 16 24 5	39.39 33.33 9.69 14.54 3.03
Clinical stage I II III IV	9 45 59 52	5.45 27.27 35.75 31.51
Site of metastasis Liver Lung Peritoneum Other	32 23 13 6	19.39 13.93 7.87 3.63
Treatment modality Curative surgery(Laparoscopic/open/emergency) Adjuvant chemotherapy Neoadjuvant chemoradiotherapy Palliative chemotherapy Palliative surgery Palliative radiotherapy	113 (16/87/10) 36 36 17 14 10	68.48 (9.69/52.72/6.06) 21.81 21.81 10.3 8.48 6.06
Defaulters Adjuvant chemotherapy Neoadjuvant chemoradiotherapy Palliative chemotherapy	82 52 0 30	49.69 31.51 0 18.18
Lost to follow up	22	13.33

*Anaemia—Haemoglobin <12 g/dl,$ anorexia and weight loss >10% of body weight in last 3 months, #emergency presentation as intestinal obstruction and perforation 1-well Differentiated Adenocarcinoma, 2- Moderately Differentiated adenocarcinoma, 3- Poorly differentiated adenocarcinoma, 4 - Others, which includes adenosquamous and squamous carcinoma

**Table 2.** One/three/five year OS.

Stage	Number	Lost to follow up	1 year (%)	3 year (%)	5 year (%)
All stages	165	22	93	81.81	30.76
I	9	1	100	100	87.5
II	45	3	100	100	83.33
III	59	13	97.8	86.95	6.52
IV	52	5	82.97	51.06	0

**Table 3.** One / three / five year DFS.

Stage	Number	Lost to follow up	1 year (%)	3 years (%)	5 years (%)
All stages	113	17	97.91	77.08	28.72
I	9	1	100	100	87.5
II	45	3	100	97.61	52.38
III	59	13	95.65	54.34	0

**Table 4.** OS as per clinical stage in months (excluding lost to follow up patients).

Stage	Frequency	Mean	Median	SD	Q1	Q3	95% CI
Over all	143	45.13	48	21	28	63	41.65–-48.62
Stage I+II+III	96	56.4	57	15.3	47.75	67	53.29–-59.5
IV	47	22.13	25	8.52	14.5	28.5	19.62–-24.63
III	42	44.65	46.5	12.19	39.25	52.75	41.03–-48.27
II	46	66.33	66	7.69	61.25	71.75	63.94–-68.73
I	8	71.75	74.5	10.47	66.75	79.25	62.99–-80.51

**Table 5.** Multiple regression analysis for factors predicting OS.

Name	Hazard ratio	*p*	Lower 95% CI	Upper 95% CI
Age	1	0.508	0.98	1.01
Sex	1.07	0.77	0.69	1.66
Smoker	0.86	0.693	0.44	1.72
Alcohol	0.29	0.409	0.67	2.68
BMI	0.80	0.598	0.36	1.81
Anaemia	0.46	0.016	0.25	0.87
Site	0.86	0.144	0.71	1.05
Hpe	1	0.982	0.83	1.2
Stage	10.1	<.001	5.82	17.98
Liver mets	1.78	0.091	0.91	3.47
Defaulters	1.08	0.718	0.71	1.64

**Table 6.** Multiple regression analysis for factors predicting DFS.

Name	Hazard ratio	*p*	Lower 95% CI	Upper 95% CI
Age	0.99	0.35	0.97	1.01
Sex	1.32	0.394	0.69	2.53
Smoker	2.45	0.838	0.48	2.49
Alcohol	1.06	0.89	0.46	2.47
BMI	0.44	0.235	0.12	1.69
Anaemia	1.91	0.05	1	3.67
Site	0.98	0.895	0.78	1.25
Hpe	1.05	0.62	0.86	1.29
Stage	19.68	<.001	5.45	71.81
Defaulters	0.73	0.304	0.41	1.32

**Table 7.** Follow up data (*N* = 143) on anaemia, BMI<18.5 and death.

Parameter	0 month	12months	24 months	36 months	48 months	60 months
Anaemia	116 (81.1%)	97 (69.3%)	68 (56.2%)	21 (23.1%)	26 (36.6%)	0 (0%)
BMI<18.5	76 (53.1%)	65 (46.4%)	46 (38.1%)	23 (25.3%)	20 (28.2%)	5 (21.8%)
Death	0	3	19	52	72	120
